# Novel Magnesium Nanocomposite for Wire-Arc Directed Energy Deposition

**DOI:** 10.3390/ma17020500

**Published:** 2024-01-20

**Authors:** Hajo Dieringa, Maria Nienaber, Danai Giannopoulou, Jonas Isakovic, Jan Bohlen, Milli Suchita Kujur, Noomane Ben Khalifa, Thomas Klein, Stefan Gneiger

**Affiliations:** 1 Institute of Material and Process Design, Helmholtz-Zentrum Hereon, Max-Planck-Str. 1, 21502 Geesthacht, Germany; maria.nienaber@hereon.de (M.N.); danai.giannopoulou@hereon.de (D.G.); jonas.isakovic@hereon.de (J.I.); jan.bohlen@hereon.de (J.B.); milli.kujur@hereon.de (M.S.K.); noomane.ben_khalifa@hereon.de (N.B.K.); 2 Institut für Produkt- und Prozessinnovation (PPI), Leuphana Universität Lüneburg, Universitätsallee 1, Gebäude 12, 21335 Lüneburg, Germany; 3 LKR Light Metals Technologies, AIT Austrian Institute of Technology, Lamprechtshausenerstraße 61, 5282 Braunau am Inn—Ranshofen, Austria; thomas.klein@ait.ac.at (T.K.); stefan.gneiger@ait.ac.at (S.G.)

**Keywords:** wire-arc directed energy deposition, nanocomposite, magnesium alloy, AM60, microstructure, mechanical properties

## Abstract

Magnesium alloys play an essential role in metallic lightweight construction for modern mobility applications due to their low density, excellent specific strength, and very good castability. For some years now, degradable implants have also been made from magnesium alloys, which, thanks to this special functionality, save patients a second surgery for explantation. New additive manufacturing processes, which are divided into powder-based and wire-based processes depending on the feedstock used, can be utilized for these applications. Therefore, magnesium alloys should also be used here, but this is hardly ever implemented, and few literature reports exist on this subject. This is attributable to the high affinity of magnesium to oxygen, which makes the use of powders difficult. Therefore, magnesium wires are likely to be used. In this paper, a magnesium-based nanocomposite wire is made from an AM60 (Mg-6Al-0.4Mn) (reinforced with 1 wt% AlN nanoparticles and containing calcium to reduce flammability), using a high-shear process and then extruded into wires. These wires are then used as feedstock to build up samples by wire-arc directed energy deposition, and their mechanical properties and microstructure are examined. Our results show that although the ductility is reduced by adding calcium and nanoparticles, the yield strength in the welding direction and perpendicular to it is increased to 131 MPa.

## 1. Introduction

Magnesium alloys have been used for many decades in aerospace, the automotive industry, and, more recently, in implantology as degradable implant materials. They have many advantages; for example, their castability is excellent, their specific strength is very high and their low Young’s modulus is very close to that of bone, which makes their application as implant materials very promising. Magnesium products are mostly processed by high-pressure die casting (HPDC); the amount of magnesium-based wrought materials is rather small.

For some years now, additive manufacturing processes have been experiencing an increased interest in academia and industry; various metallic materials are being used, but magnesium alloys play only a minor role. This is mainly due to the high affinity of magnesium to oxygen, which makes powder bed fusion (PBF) methods very difficult and potentially hazardous [1]. Hence, processes that use wires as the feedstock material for additive manufacturing are preferred due to the ease of use and economic benefits of the wires. The low wire surface area, in comparison to powder, reduces potential reactions with oxygen. These wire-based processes can be differentiated based on the method employed to melt the wire [2]. There are three main techniques for achieving this: direct melting using a laser (LDED) [3,4], by means of an electron beam (EBM) [4], or via an arc, which can be electric or plasma-based (wire-arc directed energy deposition—WADED). In WADED with electric arc melting, a wire is fed through a welding torch, and an electric current is applied. In many instances, gas metal arc welding or variants such as cold metal transfer are used for deposition [5]. In the latter process variant, the wire tip touches the conducting workpiece in a repetitive manner. Then, a short circuit is created, and an electric arc develops during the retracting motion. The accompanying heat input melts the wires’ tip, forming a droplet, which can then be deposited according to a predefined path yielding three-dimensional structures and objects [6,7]. As the welding torch typically incorporates a local gas shielding to prevent the contact of oxygen with the molten pool, the need for an enclosed fabrication chamber is eliminated for many different materials. The applicability of these processes to standard magnesium alloys has been demonstrated in the literature [5,8].

This paper investigates wire-arc directed energy deposition using a wire made of a magnesium-based nanocomposite, which, in addition to improved mechanical properties, is also characterized by reduced flammability.

## 2. Materials and Methods

### 2.1. Casting 

**Figure 1:**
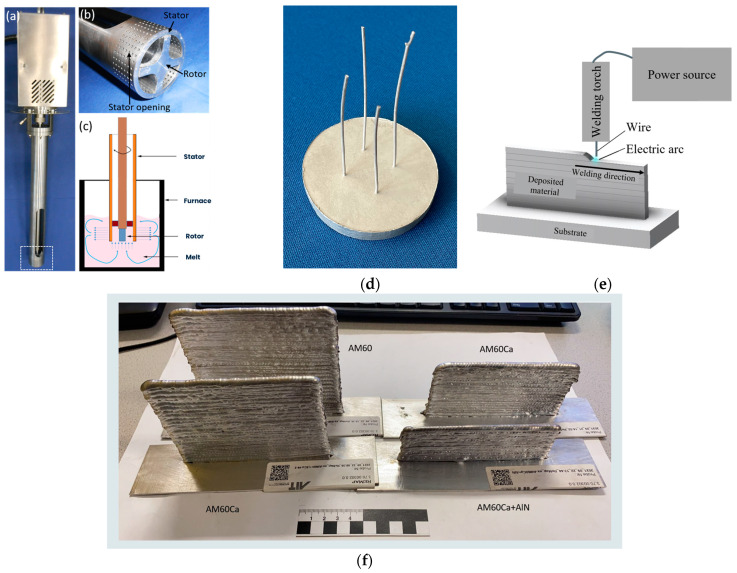
(**a**) High-shear device; (**b**) immersing rotor–stator tip of the unit; (**c**) sketch of the operating principle of the rotor–stator unit; (**d**) extrusion remnant with four wires; (**e**) schematic representation of the wire-arc directed energy deposition process [12]; (**f**) walls prepared by wire-arc directed energy deposition.

The matrix alloy selected for the nanocomposite was the magnesium alloy AM60, which is a commercially available high-pressure die casting alloy widely used in the automotive industry. Its nominal composition is Mg-6Al-0.4Mn. To reduce flammability, a second alloy was cast by adding 1.5 wt% calcium. The third material studied is the nanocomposite, in which 1 wt% AlN nanoparticles with an average particle size of about 80 nm [9] were finally added to the AM60-1.5Ca alloy. The first two alloys were heated to 720 °C in a furnace and, after stirring for 10 min, poured into cylindrical steel molds with 52 mm in diameter and 250 mm in height, which were preheated to about 400 °C. The filled ingot molds were slowly lowered at 3 mm/s into a water bath located below the ring furnace, where the ingot molds were kept at temperature. During the casting process, Ar + 1 vol% SF_6_ was used as a shielding gas. For the preparation of the nanocomposite, the AlN nanoparticles were added into the AM60+1.5Ca melt and stirred using a high-shear device. This stirring process took 10 min at a speed of 1000 rpm. The details of the process and the device used for it (Figure 1a–c) are described in more detail in [10]. Billets were then machined from the cast cylinders with a diameter of 49 mm and a length of 150 mm, which were used to extrude the wires.

### 2.2. Extrusion

The billets were heated to 350 °C, and the temperature of the container was 350 °C. An automatic extrusion press (2.5 MN max. force, Müller Engineering, Todtenweis, Germany) was used for direct extrusion; the process involved using a die with a four-fold design that allowed for the simultaneous extrusion of four wires, each with a diameter of 1 mm. A ram speed of 0.15 mm/s was set. A detailed description of the set-up and the coilers used for winding the wires is described in earlier work [11]. A careful adjustment of the drawing force during winding was required to avoid early cracking of the wires during processing, especially those with added Ca and AlN. Figure 1d shows the extrusion remainder after removing the die to visualize the high degree of deformation applied during this procedure and to reveal the feasibility of this processing approach. It is noted that the variation of wire diameter determines the geometry of the final deposited wall. With higher wire diameter, deposition rates can be improved but local shielding can become more difficult due to a larger melt pool size and lower cooling rates. Wire diameters must be chosen carefully with regard to the targeted application.

### 2.3. Ignitability

The extruded wires with a diameter of 1 mm were fixed in a screw clamp, which stands upright in a steel profile. This was set-up on a steel plate to immediately cool and solidify the dripping material. The wire was heated and melted with a propane gas flame from a GT2000 hand soldering unit (CFH, Marl, Germany), which produces a temperature of 800 °C at the flame tip. When a wire begins to burn brightly, the flame is removed to determine whether the wire continues to burn on its own, i.e., exothermically. If it merely melts, the flame continues to be directed at the wire and it continues melting.

### 2.4. Wire-Arc Directed Energy Deposition

Additive manufacturing was performed utilizing the cold metal transfer (CMT) process (Fronius, Pettenbach, Austria), and a Fronius CMT TPS power source. The torch system was handled by an ABB IRB 4600 robot with an ABB IRB 500A workpiece positioner (ABB, Zurich, Switzerland). A sheet of AZ31 (Mg-3Al-1Zn) was used as substrate material. The following parameters were applied: modified welding characteristic, welding speed of 10 mm/s in the base layer, and 6 mm/s in the building layers. Wire feed was 7 m/min in the first layer and subsequently decreased to 3.5–3.8 m/min in the building layers. Figure 1e displays a sketch of the WADED process. This resulted in a layer thickness of ca. 2.1 mm and a wall thickness of ~ 5 mm. Figure 1f shows the built-up walls. Ar + 30 vol% He was used as a cover gas during manufacturing at a flow rate of 15 l/min. To counteract the limited flowability of the melt and reduce the waviness, the welding torch was slightly oscillated.

### 2.5. Microstructure, Calcium Content, Hardness, and Mechanical Characterization

The microstructure of all the materials was observed by light optical microscopy. The specimens were embedded in a cold embedding compound (Demotec 30) and grounded with SiC abrasive papers (800–2500). The final polishing was carried out with diamond suspension and OPS (oxide polishing suspension). The polished samples were etched for 5–10 s in a standard picric acid solution of 20 mL H_2_O, 7 mL acetic acid, 200 mL ethanol, and 6 g of picric acid. For better visibility of the grain structure of the cast material, the images were taken in polarized light. SEM/EDX analysis was performed with a scanning electron microscope ZEISS Crossbeam 550 L (ZEISS, Oberkochen, Germany).

Due to the tendency of calcium-containing magnesium alloys to evaporate calcium in the molten state, an ICP-OES (Inductively Coupled Plasma–Optical Emission Spectrometry) analysis of the calcium content of the wires, the built-up walls of the AM60+1.5Ca, and the AM60+1.5Ca+1AlN_NP,_ was carried out using an Acros II FHX22 (SPECTRO Analytical Instruments GmbH, Kleve, Germany). AM60+1.5Ca was dissolved in 65% nitric acid, and AM60+1.5Ca+1AlN_NP_ was dissolved in a mixture of 37% hydrochloric acid and 65% nitric acid. Five test samples of each material were analyzed, and the mean value was given.

To determine the hardness, 10 indents were taken as an average using an EMCO test M2C010. HV5 was applied for the casting material, and HV0.5 for the wires and walls (vertical and horizontal).

Tensile tests were performed on a Z050 universal testing machine with a maximum load of 50 kN (Zwick GmbH & Co.KG, Ulm, Germany). For this purpose, the walls were milled to a thickness of 2 mm. The dogbone-shaped specimens were machined to a gauge length of 18 mm and a width of 5 mm in the vertical and horizontal directions. At least three specimens were tested to failure at a constant initial strain rate of 10^−3^ s^−1^ at room temperature for statistical validation. A mechanical extensometer was used to record the change in length of the specimen. Stress–strain diagrams were generated from the recorded data, and mechanical properties were obtained.

## 3. Results

### 3.1. Microstructure and Hardness

**Figure 2:**
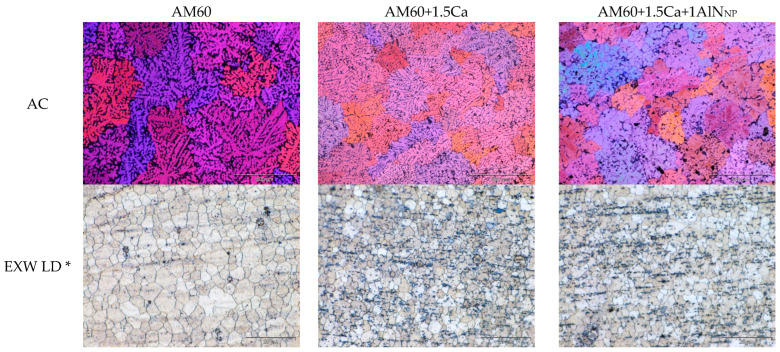

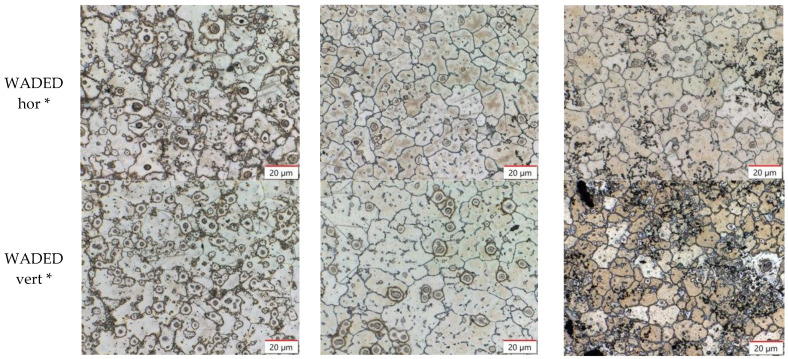
Micrographs of three investigated materials—AM60, AM60+1.5Ca, and AM60+1.5Ca+1AlN_NP_—in three different conditions: (i) as-cast (AC), (ii) extruded condition of wires (EXWs), and (iii) walls after WADED horizontal and vertical. * LD: longitudinal direction; hor: horizontal; vert: vertical. Please note: scale bar length for AC materials is 500 µm; for EXW and WADED materials it is 20 µm.

Light optical microscope images were taken and grain size measurements were performed in all three conditions of the three materials: (i) the as-cast condition (AC), (ii) the extruded condition of the wires (EXWs), and (iii) the as-deposited condition after wire-arc directed energy deposition (WADED). The microstructures of the three materials in the three states are shown in Figure 2. Table 1 summarizes the grain sizes and hardness of the respective materials in the three conditions.

In the as-cast materials, a grain refinement, in the case of the addition of calcium to AM60 of about 20%, is observed. Other studies have already observed this effect and attributed it to the formation of an Al_2_Ca phase [13,14,15]. The further addition of AlN nanoparticles to AM60+1.5Ca does not lead to any further grain refinement. A much stronger grain refining effect was observed with the combination of AM60 and AlN nanoparticles when prepared by a different process and without the addition of calcium [16]. When the AlN nanoparticles are stirred with ultrasound assistance, a reduction in grain size of 93.3% can be achieved. It is therefore hypothesized that the high-shear process does not have the effectiveness of ultrasonic casting, which may cause the lowered grain refinement. However, the presence of calcium can also lead to a reduction in the grain refinement potential of the nanoparticles by passivating the nanoparticle surface. An increase in hardness due to calcium addition, and thus the formation of Al_2_Ca precipitates, has been observed [13]. An increase in hardness due to the addition of AlN nanoparticles has already been shown in extruded materials in [17]. The results from [17], therefore, also fit the hardness and grain size of the materials investigated here.

The grain size of the extruded AM60 (5.5 µm) is somewhat reduced to 3.4 µm by the addition of calcium, while no further reduction is observed with the addition of AlN nanoparticles. Note that the variation is small and could also well be related to local fluctuations of the extrusion exit speed, which determines the dynamic recrystallization behavior to a high extent [18]. Still, the hardness of AM60-1.5Ca and AM60-1.5Ca-1AlN_NP_ is about 75 HV0.5, which is slightly higher than that of AM60 with 70.4 HV0.5 despite the rather small changes in the grain structure. This also fits with the results in [17].

The additively manufactured walls were prepared from two directions for metallography and hardness measurements to analyze the formation of columnar grains, that often occur in additive manufacturing, and their potential effects on the mechanical anisotropy: (i) a top view (hor.) and a view across the individual planes (vert.). The grain sizes are approximately identical in the case of horizontal preparation. This suggests that little solidification texture in terms of grain morphology prevails, although this is often observed in additive manufacturing due to the thermal gradient present upon solidification [19]. In the case of the vertical preparation, the grain size of the nanocomposite with 16.1 ± 1.0 µm is significantly smaller than the grain sizes of AM60 and AM60+1.5Ca with 23.7 ± 2.0 µm and 22.7 ± 3.0 µm. The hardness values increase with the addition of calcium and AlN nanoparticles. The difference between the AM60 and AM60+1.5Ca nanocomposites is about 10–12 HV5, which may be caused by the combined effects of grain refinement and particle strengthening [20].

An analysis of the calcium concentration in the wires and the built-up walls using ICP-OES resulted in a calcium concentration of 1.42 ± 0.01 wt% and 1.42 ± 0.01 wt% for AM60+1.5Ca. The nanocomposite AM60+1.5Ca+1AlN_NP_ contains 1.37 ± 0.01 wt% calcium in the wire and 1.35 ± 0.03 wt% calcium in the built-up wall. This means that calcium does not evaporate to an extent that is relevant when calculating the calcium addition during alloying. This is certainly due to the very brief duration of the molten state of the wire material before it solidified on the wall.

### 3.2. Mechanical Properties in Tensile Tests

**Figure 3:**
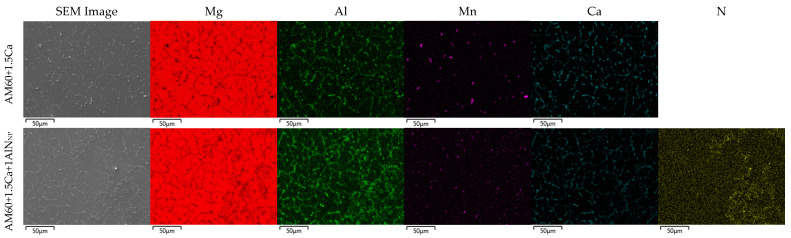
SEM BSE image and EDX mapping results for Mg, Al, Mn, Ca, and N.

Tensile tests at room temperature were performed on walls made of the three materials AM60, AM60+1.5Ca, and AM60+1.5Ca+1AlN_NP_. Samples were taken and tensile testing was conducted in the horizontal and vertical directions. The results of the tensile tests can be found in Table 2. The addition of 1.5 wt% calcium to the AM60 results in a slight decrease in yield strength. The further addition of the nanoparticles, on the other hand, leads to an increase in the yield strength, which also clearly exceeds the values of AM60. This applies to both directions, vertical as well as horizontal. The symmetry of the values of the yield strength is remarkable, especially in the case of the nanocomposite with 131 MPa in the horizontal and vertical directions, which is in line with the globular grain morphology observed in all alloys after WADED. The tensile strength and strain decreased with the addition of calcium, and they decreased further with the addition of nanoparticles. SEM mappings of the walls in the vertical direction (see Figure 3) clearly show a high amount of phases containing Al and Ca at the grain boundaries of AM60+1.5Ca and AM60+1.5Ca+1AlN_NP_. The nitrogen mapping of AM60+1.5Ca+1AlN_NP_ shows a homogeneous distribution of nanoparticles with only a few isolated agglomerates. The Al_2_Ca phase and the nanoparticles possibly act as crack initiators, and the earlier failure leads to only moderate elongations at failure and ultimate tensile strength. The presence of AlN particle agglomerates may also affect the crack propagation to an unknown extent.

### 3.3. Ignitability of the Wires

**Figure 4:**
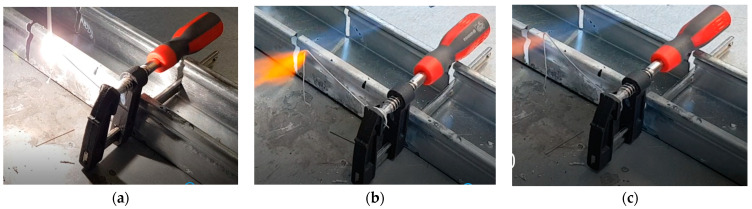
(**a**) Continuously burning AM60, (**b**) melting, but not burning, AM60-1.5Ca, and (**c**) AM60-1.5Ca-1AlN_NP_.

Wires made of the three materials AM60, AM60+1.5Ca, and AM60+1.5Ca+1AlN_NP_ were heated beyond their melting temperature using a propane gas flame to test their high-temperature oxidation behavior. A significantly different behavior of the wire made of AM60 and the two wires containing additional calcium was observed. The AM60 wire began to burn brightly immediately after melting. The bright white flame, which can be seen in Figure 4a, did not self-extinguish even when the gas flame was removed. The wire continued burning with this bright flame until its fixation in the screw clamp. The AM60+1.5Ca and AM60+1.5Ca+1AlN_NP_ wires, on the other hand, did not ignite; they merely melted and the melting stopped as soon as the flame was removed (see Figure 4b,c). Only when heated further did the melting start again. The molten material dropped onto the steel plate and solidified without any further burning.

It is known that the addition of calcium to magnesium alloys increases the flammability resistance [21,22,23], which could also be demonstrated here. Even with a small amount of Ca addition, an additional oxide layer composed of calcium oxide (CaO) can form on the surface of Mg. This CaO layer is less porous than the native MgO layer, serving as an effective barrier against oxygen diffusion. The further addition of AlN showed no deterioration in the high-temperature oxidation behavior of the AM60+1.5Ca wire. In the specific wire-based additive manufacturing process used in this work, the molten bath is protected by inert gas; therefore, Ca addition does not directly impact the manufacturing process itself. Nevertheless, there are notable benefits associated with alloy production, handling, and application.

## 4. Discussion

**Figure 5:**
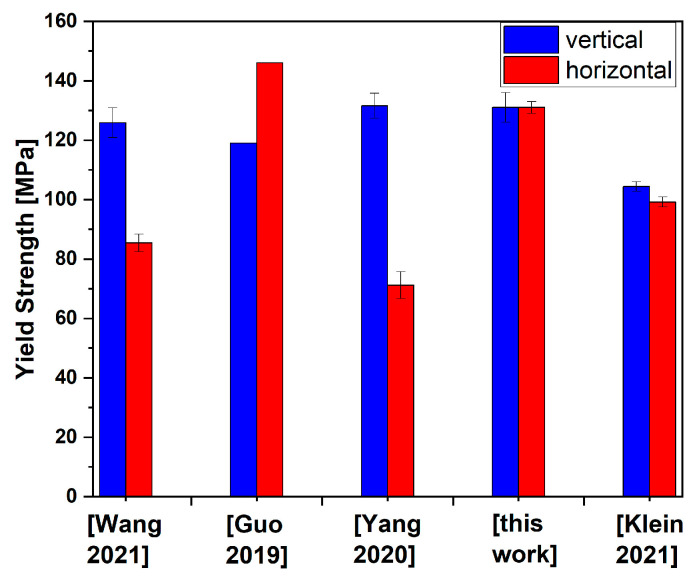
Yield strength values determined in vertical and horizontal directions in Klein 2021 [5], Wang 2021 [24], Guo 2019 [25] and Yang 2020 [26] and this paper.

Comparable investigations on magnesium alloys have shown a strong anisotropy of the yield strengths in both directions of the built-up plane. Wang et al. [24] investigated a wall built up from AZ31, also using the WADED process, in order to determine the optimal process parameters. A yield strength of 85.4 MPa was found in the horizontal direction and 125.9 MPa in the vertical direction. This also showed an anisotropy in the microstructure, which explains the anisotropy of the yield strength. Guo et al. [25] determined the yield strengths of a WADED-processed wall made of AZ80M in the horizontal and vertical directions to be 146 MPa and 119 MPa, respectively. This anisotropy was also explained by the inhomogeneous microstructure. In another investigation on AZ31 walls fabricated using WADED, it is again shown that the yield strengths in the horizontal and vertical directions are very different in the built-up plane [26]. They were found to be 71.2 MPa and 131.6 MPa, respectively. In [26], it is proposed to define the in-plane anisotropy as (%IPA) = (X_max_ − X_min_)/X_max_. In [24,25,26], this resulted in %IPA values of 0.32, 0.18, and 0.46, respectively, which corresponds to high anisotropy. Meanwhile, in our study, we observed identical yield strength in the horizontal and vertical directions, and thus %IPA = 0. A comparably low in-plane anisotropy of 0.05 has so far only been observed in [5] in an AZ61 magnesium alloy, although at a lower level. Yield strength values in the vertical and horizontal directions determined in [5,24,25,26] and this paper are shown in Figure 5.

The addition of ceramic nanoparticles to magnesium or aluminum alloys is generally used to increase the mechanical properties and, in most cases, yield strength [27]. This increase in yield strength can be achieved by different mechanisms. A direct-acting mechanism is Orowan strengthening, where the particles restrict the dislocation movement [28]. Another mechanism is grain refinement, which plays a role, especially in cast materials, and can thus be relevant in this case when, after melting the wires, the drop solidifies. We have already shown in [16] that other strengthening mechanisms, such as the creation of geometrically necessary dislocations caused by differences in the coefficient of thermal expansion, the Young’s moduli, or the load-bearing mechanism, do not make significant contributions to the strengthening. Consequently, in the following, we will only consider the Orowan mechanism and grain refinement, whose influence on the strength can be described by the Hall–Petch relationship [29,30]. The amount of Orowan strengthening $\Delta \sigma_{OR}$ is expressed according to [28] by Equation (1), where *b* is the Burgers vector (=0.32 nm), $V_{p}$ is the volume fraction of 1 wt% AlN nanoparticles (≈5.5214 × 10^−3^), $G_{m}$ is the shear modulus of the matrix alloy (≈11.6 GPa), and $d_{p}$ is the average diameter of the AlN nanoparticles (≈80 nm). According to these assumptions, the contribution of the Orowan strengthening to the total strengthening is calculated to be 11.9 MPa. The strengthening by grain refinement, which is also called Hall–Petch strengthening or grain boundary strengthening, follows Equation (3), from which, in turn, Equation (4) follows, assuming the identical manufacturing processes of the materials. Since the AlN nanoparticles have been added to the AM60+1.5Ca and the influence of the nanoparticles on the grain refinement is to be calculated, we chose the mean value of the horizontal and vertical yield strength of the calcium-containing AM60 (=114.5 MPa) and the nanocomposite (=131 MPa) from Table 1, for the calculation. Under this assumption and with *k_y_* = 530.2 MPa*µm ^½^ [16], the contribution to the increase in strength by grain refinement of 7.4 MPa is calculated. The sum of the Orowan and Hall–Petch contributions equals 19.3 MPa, while our experiments showed a difference of 16.5 MPa. Assuming that there is no ideal distribution of nanoparticles in a metallic matrix, the theory seems to fit well with the yield strengths of the materials investigated in this study.

(1) $$\Delta \sigma_{OR}=\frac{0.13bG_{m}}{\lambda }ln\frac{d_{p}}{2b}$$

where

(2) λ=dp12Vp13−1

(3) $$\sigma_{y}=\sigma_{0}+k_{y}D^{-1/2}$$

(4) $$\Delta \sigma_{GR}=k_{y}\left(\frac{1}{\sqrt{D_{MMNC}}}-\frac{1}{\sqrt{D_{0}}}\right)$$

## 5. Conclusions

The manufacturing of a magnesium-based nanocomposite wire, based on a non-flammable magnesium alloy AM60+1.5Ca for use in additive manufacturing, was successfully shown in this study. Firstly, the method of producing the nanocomposite using a high-shear process and then the extrusion of the material to form wires has been shown to be feasible. The wire-based WADED process, by which the walls were built, is able to process the nanocomposite. Calcium does not significantly evaporate during the WADED process, as the duration of the liquid state is insufficient. The nanoparticles show a homogeneous distribution in the built-up structures and contribute to an increase in strength in two ways: (i) by Orowan strengthening and (ii) by grain refinement. The isotropy of the yield strength in the vertical and horizontal directions is remarkable—potentially enabling ease of structural/mechanical design in actual components. In both cases, a very good yield strength of 131 MPa is achieved.

## Figures and Tables

**Table 1:** Grain sizes (GS) and hardness (HV5 or HV0.5) of three investigated materials—AM60, AM60+1.5Ca, and AM60+1.5Ca+1AlN_NP_—in three different conditions: (i) as-cast (AC), (ii) extruded condition of wires (EXWs), and (iii) walls after wire-arc directed energy deposition (WADED horizontal and vertical).

	AM60	AM60+1.5Ca	AM60+1.5Ca+1AlN_NP_
AC	GS: 457 ± 24 µm	GS: 363 ± 18 µm	GS: 372 ± 16 µm
	HV5: 46.3 ± 2.2	HV5: 54.1 ± 2.8	HV5: 51.9 ± 8.9
EXW LD *	GS: 5.5 ± 0.3 µm	GS: 3.4 ± 0.1 µm	GS: 4.0 ± 0.1 µm
	HV0.5: 70.4 ± 3.5	HV0.5: 75.2 ± 2.2	HV0.5: 74.8 ± 1.7
WADED hor *	GS: 18.4 ± 1.0 µm	GS: 18.1 ± 2.0 µm	GS: 18.3 ± 2.0 µm
	HV0.5: 50.0 ± 1.0	HV0.5: 60.8 ± 2.3	HV0.5: 62.3 ± 2.2
WADED vert *	GS: 23.7 ± 2.0 µm	GS: 22.7 ± 3.0 µm	GS: 16.1 ± 1.0 µm
	HV0.5: 50.0 ± 1.3	HV0.5: 54.2 ± 1.1	HV0.5: 60.4 ± 2.0

* LD: longitudinal direction; hor: horizontal; vert: vertical.

**Table 2:** Mechanical properties of the walls of the three investigated materials AM60, AM60+1.5Ca, and AM60+1.5Ca+1AlN_NP_ in the vertical and horizontal directions after wire-arc directed energy deposition tested in tension at room temperature.

Material	YS [MPa]	UTS [MPa]	ε_f_ [%]
AM60—vertical	121 ± 2	266 ± 5	10.9 ± 0.8
AM60—horizontal	126 ± 13	262 ± 4	9.9 ± 1.0
AM60+1.5Ca—vertical	116 ± 6	236 ± 4	6.5 ± 0.6
AM60+1.5Ca—horizontal	113 ± 5	221 ± 12	4.1 ± 1.3
AM60+1.5Ca+1AlN_NP_—vertical	131 ± 5	226 ± 4	4.3 ± 0.8
AM60+1.5Ca+1AlN_NP_—horizontal	131 ± 2	219 ± 8	3.1 ± 0.5

## Data Availability

Data are contained within the article.
